# Transcutaneous carbon dioxide measurements in anesthetized apneic patients with BMI > 35 kg/m^2^

**DOI:** 10.1007/s00540-023-03263-8

**Published:** 2023-10-10

**Authors:** Victoria A. Eley, Louis Guy, Christine Woods, Stacey Llewellyn, Andre A. J. Van Zundert

**Affiliations:** 1 Department of Anaesthesia and Perioperative Medicine, Royal Brisbane and Women’s Hospital, Butterfield St, Herston, QLD 4029 Australia; 2 Faculty of Medicine, The University of Queensland, Herston Road, Herston, QLD 4006 Australia; 3 Statistics Unit, QIMR Berghofer Medical Research Institute, Herston Road, Herston, QLD 4006 Australia; 4 Department of Anaesthesia and Perioperative Medicine, Royal Brisbane and Women’s Hospital, Butterfield St, Herston, Brisbane, 4006 Australia

**Keywords:** Apneic oxygenation, Carbon dioxide, High flow nasal oxygen, Obesity, Transcutaneous monitoring

## Abstract

Transcutaneous carbon dioxide measurement (TcCO_2_) offers the ability to continuously and non-invasively monitor carbon dioxide (CO_2_) tensions when end-tidal monitoring is not possible. The accuracy of TcCO_2_ has not been established in anesthetized apneic patients with obesity. In this secondary publication, we present a methods comparison analysis of TcCO_2_ with the gold standard arterial PCO_2_, in adult patients with body mass index (BMI) > 35kg/m^2^ who were randomized to receive high flow or low flow nasal oxygenation during post-induction apnea. Agreement between PaCO_2_ and TcCO_2_ at baseline, the start of apnea and the end of apnea were assessed using a non-parametric difference plot. Forty-two participants had a median (IQR) BMI of 52 (40–58.5) kg/m^2^. The mean (SD) PaCO_2_ was 33.9 (4.0) mmHg at baseline and 51.4 (7.5) mmHg at the end of apnea. The bias was the greatest at the end of apnea median (95% CI, 95% limits of agreement) 1.90 mmHg (−2.64 to 6.44, −7.10 to 22.90). Findings did not suggest significant systematic differences between the PaCO_2_ and TcCO_2_ measures. For a short period of apnea, TcCO_2_ showed inadequate agreement with PaCO_2_ in patients with BMI > 35 kg/m^2^. These techniques require comparison in a larger population, with more frequent sampling and over a longer timeframe, before TcCO_2_ can be confidently recommended in this setting.

## Introduction

Transcutaneous CO_2_ monitoring (TcCO_2_) has been shown to give a reliable estimation of the arterial partial pressure of carbon dioxide (PaCO_2_) in healthy volunteers [1]. The use of high-flow nasal oxygen delivery facilitates short laryngeal procedures but risks carbon dioxide (CO_2_) retention during apnea and precludes accurate end-tidal CO_2_ monitoring. TcCO_2_ monitoring offers a potential benefit in this context but has not been studied in apneic obese patients.

Studies comparing the accuracy and precision of TcCO_2_ with PaCO_2_ in patients with body mass index (BMI) > 35 kg/m^2^ in a perioperative setting have reported good accuracy and precision [2] when compared against the accepted clinical range of agreement ± 7.5 mmHg [3]. In this secondary publication, we present data obtained during a study of patients with BMI > 35 kg/m^2^, comparing safe apnea time between those administered high flow nasal oxygen and those with standard nasal prongs [4]. We aimed to measure the agreement of CO_2_ measurements obtained from arterial blood samples (PaCO_2_) and transcutaneous monitoring (TcCO_2_) at three time points in obese patients under apneic conditions.

## Methods

This report comprises a secondary publication based on additional data that were collected during a single-center randomized controlled trial undertaken between September 2018 and May 2019 at the Royal Brisbane and Women’s Hospital [4]. The trial was ethics-approved (HREC-18-QPCH-9 approved 21-03-2018), registered (ANZCTR 12618000445279) and participants provided written informed consent. Measurement of TcCO_2_ was stated in the trial registration and included in the initial ethical approval. We tested the hypothesis that the use of Optiflow THRIVE™ (Fisher & Paykel Healthcare, Auckland, New Zealand) at 70 L/min would increase the time to hypoxia and increase carbon dioxide elimination in apneic obese patients, when compared with 4 L/min oxygen administered via standard nasal prongs. The 42 participants were undergoing laparoscopic bariatric surgery, had BMI ranging from 38 to 68 kg/m^2^ and a mean (SD) PaCO_2_ at the end of apnea of 51.4 mmHg (7.5) (range 27.2–64.8 mmHg). The full inclusion and exclusion criteria and detailed methodology are available [4].

All participants were monitored according to local standards, with the addition of invasive radial arterial catheters. Participants were randomized to Group T, in which they were administered high flow nasal oxygen using Optiflow THRIVE™ at 70 L/min, humidified using the Fisher & Paykel 950 humidifier, providing 70% relative humidity (oxygen concentration delivered at 100%); or to Group N, in which they were administered standard nasal prongs at 4 L/min (Salter Labs, Arvin, CA, USA). In both groups, pre-oxygenation occurred in the ramped position and with FiO_2_ 1.0 at 10 L/min.

Induction medications consisted of an opioid, propofol, and rocuronium with dosing at the discretion of the anesthetist. Anesthesia maintenance was achieved using propofol target controlled infusion. The patients were bag-mask ventilated to achieve an end-tidal oxygen fraction > 0.9. At this point, nasal oxygenation was applied according to group allocation and airway patency maintained using an oropharyngeal airway and two-handed airway maneuvers (chin lift, head tilt, jaw thrust).

Clinical observations and sampling occurred while the participants were anesthetized and apneic. TcCO_2_ was measured using the TCM5 FLEX transcutaneous monitor TC Sensor 92 and 32mm TOSCA fixation ring (Radiometer Pacific Pty. Ltd., Waverley, Victoria, Australia). The device sensor was calibrated for each patient, to reach the required temperature of 43.5 degrees Celsius. The participant’s skin was prepared with 70% isopropyl alcohol prior to placing the fixation ring over the clavicle and a small amount of contact gel applied before sensor attachment. Sensor membranes were replaced every three days. Arterial blood gas samples and TcCO_2_ measurements were obtained at baseline (prior to induction to anesthesia), at the start of apnea (T0), and the end of apnea (Tend). Tend was defined as the time at which peripheral arterial oxygen saturations (measured by pulse oximetry) dropped to ≤ 95% or a total of 360s of apnea, whichever occurred first. Participant characteristics were collected and these included age, sex, BMI, and co-morbidities including obstructive sleep apnea.

The sample size was a convenience sample of 42, determined according to the primary outcome of the original study [4]. The original analyses demonstrated no statistical differences between Group T and Group N in terms of the median PaCO_2_ post apnea or the rate of increase of PaCO_2_ during apnea. Therefore, for this methods comparison analysis, 42 participants were analyzed as one group. Agreement between PaCO_2_ and TcCO_2_ at the three time points was assessed using a non-parametric difference plot. Bias was reported as the median of the mean differences for each participant, with 95% confidence intervals (CI). The 95% limits of agreement (LOA) were estimated by the 2.5th and 97.5th percentiles with 90% CI, using quantile regression with cluster-robust bootstrap inference [5–7]. Analyses were run in R statistical package version 4.1.3.

## Results

42 participants had a mean (SD) age of 50.4 (11.1) years, 33 (78.6%) were female and they had a median (IQR) BMI of 52 (40–58.5) kg/m^2^. Twenty-two (52.4%) were diagnosed with obstructive sleep apnea. Additional characteristics are reported elsewhere.^4^ In 14 (33.3%) participants, the apneic period lasted ≥ 360s. In the remaining 28, the mean (SD) time to desaturation to ≤ 95% (apneic period) was 190.3s (73.7). The mean (SD) PaCO_2_ was 33.9 (4.0) mmHg at baseline and 51.4 (7.5) mmHg at the end of apnea.

**Fig. 1:**
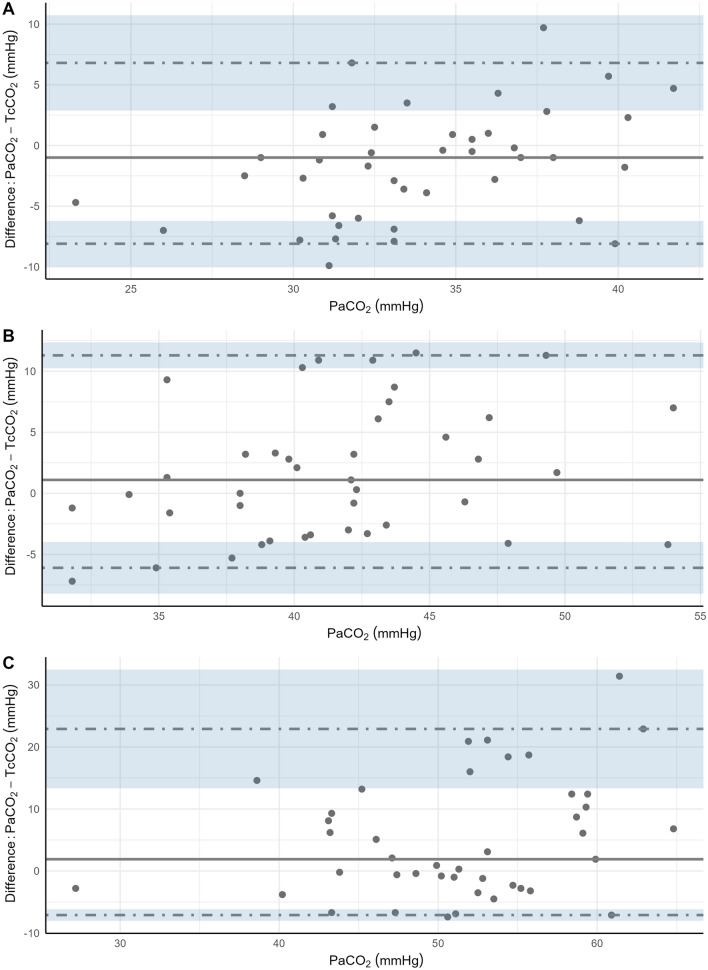
Difference plot of PaCO_2_ and TcCO_2_ at **A** baseline, **B** start of apnea **C** end of apnea, showing bias estimated as the median of the differences (solid black line) and dashed lines displaying non-parametric 95% limits of agreement estimated using the 2.5th and 97.5th percentiles with 90% confidence intervals

**Fig. 2:**
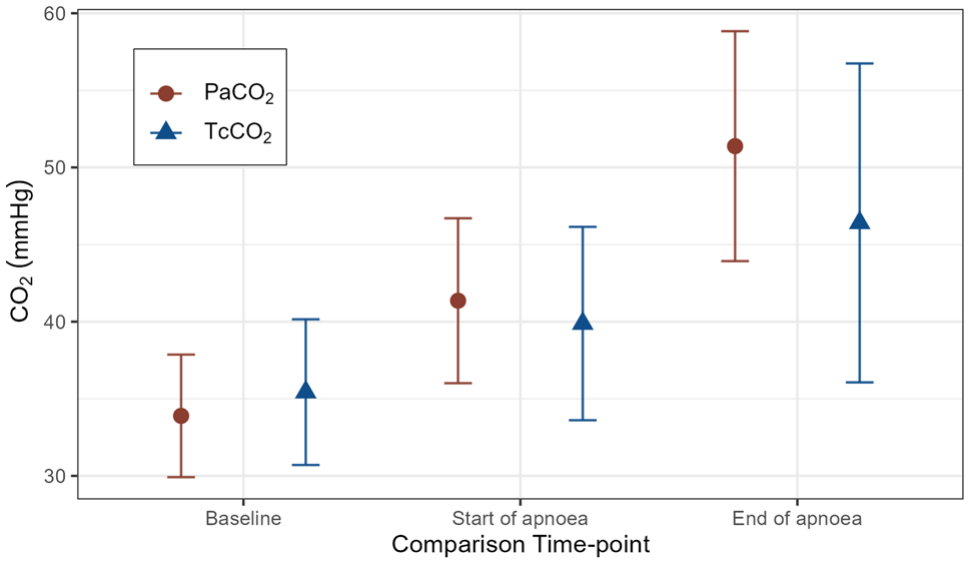
PaCO_2_ and TcCO_2_ measures at the three time points. Values are mean (SD). PaCO_2_ data have been published previously [4]

The bias (95% CI, 95% LOA) at baseline was −1.00 mmHg (−2.47 to 0.47, −8.10 to 6.80), at T0 was 1.10 mmHg (−1.52 to 3.72, −6.10 to 11.30), and at Tend was 1.90 mmHg (−2.64 to 6.44, −7.10 to 22.90). Figure 1 shows the difference between the measurement techniques for each time point. The area between the limits of agreement indicates where 95% of the differences are expected to occur. Compared to PaCO_2_, TcCO_2_ had a small upward bias (measurement greater than PaCO_2_) at baseline and small downward bias (measurement lower than PaCO_2_) at time points T0 and Tend (Fig. 2). However, the 95% CI for the bias estimates all contain zero, suggesting no significant systematic differences between the PaCO_2_ and the TcCO_2_ measures.

## Discussion

To the best of our knowledge, these are the first published data describing TcCO_2_ monitoring in apneic patients with obesity. Our results suggest that for a short period of apnea, TcCO_2_ monitoring may be inaccurate when used to estimate PaCO_2_ in patients with BMI > 35 kg/m^2^. The bias LOA were outside the recommended range of ± 7.5 mmHg, with wide 90% CIs at the start and end of apnea. The accuracy of TcCO_2_ in anesthetized apneic patients has been previously reported, but in populations with normal BMI [8, 9]. Measuring TcCO_2_ over longer apneic periods (median of 14 min [9] and up to 45 min [8]), these studies showed conflicting results. Based on Bland–Altman analysis, Pape et al. reported acceptable agreement, with a tendency for TcCO_2_ to overestimate CO_2_ tensions after 15 min of apnea, when PaCO_2_ levels were the highest [9]. Schweizer et al. concluded these two methods were not interchangeable [8]. Their linear mixed model demonstrated a substantial bias of −19.1mmHg (95% CI −20.1 to −18.0) between arterial and TCM5 measurements [8].

TcCO_2_ measurements offer a convenient and non-invasive method of detecting hypercapnia in non-obese, anesthetized, apneic patients. There remain limitations of the method that must be considered [3]. Correct calibration is required and should be undertaken according to manufacturer recommendations. Previous evaluations of TcCO_2_ measurements in patients with obesity have placed the sensor on the forearm to avoid increased subcutaneous tissue [2], whereas we placed the sensor over the clavicle. Heating of the electrode prior to placement may be recommended by the manufacturer and this can lead to thermal injury of the skin, whereas failure to heat the electrode may cause invalid results. While our participants were demonstrably hypercapnic, our results pertain to a small number of samples from a relatively small population and over a short timeframe. Conditions of prolonged apnea in this population will be challenging to reproduce from an ethical and safety perspective. These measurement techniques should be evaluated in a larger population, with more frequent sampling over a longer timeframe, before TcCO_2_ measurement can be confidently recommended for patients with obesity under apneic conditions.

## Data Availability

Data are available on reasonable request, from the authors.
